# C-Reactive Protein-to-Albumin Ratio as Prognostic Marker for Anal Squamous Cell Carcinoma Treated With Chemoradiotherapy

**DOI:** 10.3389/fonc.2019.01200

**Published:** 2019-11-08

**Authors:** Daniel Martin, Franz Rödel, Panagiotis Balermpas, Ria Winkelmann, Emmanouil Fokas, Claus Rödel

**Affiliations:** ^1^Department of Radiotherapy and Oncology, University of Frankfurt, Frankfurt, Germany; ^2^Frankfurt Cancer Institute (FCI), Frankfurt, Germany; ^3^German Cancer Research Center (DKFZ), Heidelberg, Germany; ^4^Partner Site Frankfurt am Main, German Cancer Consortium (DKTK), Frankfurt, Germany; ^5^Department of Radiation Oncology, University Hospital Zürich, Zurich, Switzerland; ^6^Senckenberg Institute for Pathology, University of Frankfurt, Frankfurt, Germany

**Keywords:** anal cancer, C-reactive protein, albumin, biomarker, disease-free survival

## Abstract

**Background:** Definitive chemoradiotherapy (CRT) is the primary treatment for non-metastatic anal squamous cell carcinoma (ASCC). Despite favorable treatment outcomes in general, failure rates up to 40% occur in locally advanced disease. For treatment escalation or de-escalation strategies easily assessable and valid biomarkers are needed.

**Methods:** We identified 125 patients with ASCC treated with standard CRT at our department. C-reactive protein (CRP) to albumin ratio (CAR) was calculated dividing baseline CRP by baseline albumin levels. We used maximally selected rank statistics to dichotomize patients to high and low risk groups. Associations of CAR with clinicopathologic parameters were evaluated and the prognostic impact was tested using univariate and multivariate cox regression analysis. In a subset of 78 patients, pretreatment tumor tissue was available and CD8+ tumor infiltrating lymphocytes (TILs) and p16^INK4a^ status were scored by immunohistochemistry and correlated with CAR.

**Results:** Advanced T-stage and male gender were significantly associated with higher baseline CAR. Using the calculated cutoff of 0.117, a high baseline CAR was also associated with worse locoregional control (*p* = 0.002), distant metastasis-free survival (*p* = 0.01), disease-free survival (DFS, *p* = 0.002) and overall survival (OS, *p* < 0.001). A combined risk score incorporating N-stage and CAR, termed N-CAR score, was associated with worse outcome across all endpoints and in multivariate analysis independent of T-stage and Gender (HR 4.27, *p* = 0.003). In the subset of 78 patients, a strong infiltration with intratumoral CD8+ TIL was associated with a significantly lower CAR (*p* = 0.007). CAR is an easily accessible biomarker that is associated with DFS. Our study revealed a possible link between chronic systemic inflammation and an impaired intratumoral immune response.

## Introduction

Anal squamous cell carcinoma (ASCC) is characterized by increasing incidence in developed countries (1, 2). Chemoradiotherapy (CRT) is the primary treatment for non-metastatic disease (3). In general, treatment outcomes are favorable but relapse occurs in up to 40% of patients with advanced stages (cT3-4 and/or cN+) (3, 4). Pretreatment biomarkers that can be obtained easily are needed to select patients for treatment escalation strategies. Conversely, as CRT can be associated with substantial long-term gastrointestinal toxicities (5), there is a need for de-escalation strategies to avoid overtreatment in patients with favorable prognosis. Randomized trials have established prognostic clinical factors like T-stage, N-stage and gender (6, 7). More recently, human papilloma virus (HPV)/p16^INK4a^ status and infiltration with CD8+ tumor infiltrating lymphocytes (TIL) have been validated as prognostic molecular markers (8–11).

The role of the immune system for cancer progression has been elucidated in recent decades. The prognostic impact of inflammation-associated blood cells, like leukocytes or neutrophils, has been reported in ASCC (12–14) and other malignancies (15, 16). C-reactive protein (CRP) is an acute phase protein, secreted by the liver upon interleukin 6 (IL-6) stimulation, and physiologically acts as part of the complement system (17). Clinically, CRP is used mainly as a marker for inflammation. Patients with colorectal cancer display significantly elevated baseline CRP levels as compared to healthy individuals (18).

Serum albumin levels can be an indicator of malnutrition that in turn is associated with impaired immune response in cancer patients (19). Due to their close link, the ratio of CRP to albumin (CAR) has been proposed as a marker to identify patients at risk for early mortality from sepsis (20). Moreover, high baseline CAR values have been associated with worse prognosis in head and neck squamous cell, bladder, esophageal cancer, and others (21–24), but have not been evaluated in ASCC yet. In the present study, we aimed to investigate the correlation of CAR with established clinical (T/N-stage, gender) and molecular prognostic factors (CD8+ TIL, p16INK4a) and analyze its prognostic value in patients with ASCC treated with standard definitive CRT.

## Materials/Methods

### Patients and Treatment Protocol

We identified 126 patients with histologically confirmed, non-metastatic ASCC treated between 12/2004 and 12/2016 with definitive CRT at our department. Written consent and approval from the institutional review board and patients was obtained. Patients were routinely staged by physical and rectal-digital examination, proctoscopy with biopsy, CT or MRI of the abdomen and pelvis, chest X-ray, serum chemistry, and complete blood count. TNM staging was done according to the UICC version 7.

Radiotherapy was applied using either 3D-conformal radiotherapy (RT) or intensity-modulated-RT (IMRT). Patients were treated with a median total dose of 59.4 Gray (Gy) with daily fractions of 1.8 or 2 Gy. Chemotherapy was applied in the first and fifth week of radiotherapy and consisted of 5-fluorouracil (1,000 mg/m^2^/day or 800 mg/m^2^/day) as 4 or 5 day continuous infusion and mitomycin C (MMC) given as an intravenous bolus (10 mg/m^2^) on day 1 of each cycle.

### Response Assessment and Follow-Up

Initial response assessment was scheduled 8–10 weeks after completion of CRT and every 3 months afterwards for the first 2 years, followed by 6 month intervals thereafter. Examination included physical and digital rectal examination, proctoscopy (with biopsies in case of suspicious lesions) and pelvic CT/MRI scan.

### Serum Chemistry

We collected the baseline CRP and albumin values from our hospital database. Blood samples were collected as part of routine test battery before application of chemotherapy. Baseline was defined as either the day of treatment initiation or up to 4 days before. CAR was calculated by dividing CRP in mg/dl through albumin in g/dl.

### Immunohistochemistry for CD8 TIL and p16^INK4a^

In a subset of 78 patients formalin fixed paraffin embedded (FFPE) pretreatment tumor tissue was available and immunohistochemical staining of CD8 TIL and p16^INK4a^ was performed by a horseradish-peroxidase technique using a DAKO Autostainer Link 48 (DAKO, Hamburg, Germany) with standardized Dako EnVision™ FLEX Blocking reagent (K800, DAKO), CD8 antibody (1:100, clone C8/144B; Dako M7103) and a CINtec histology kit (Roche, Heidelberg, Germany) for p16^INK4a^. Next, dextran polymer conjugated horseradish-peroxidase and 3,3'-diaminobenzidine (DAB) chromogen was used for visualization and hematoxylin solution (Gill 3, Sigma Aldrich, Munich, Germany) for counterstaining. Negative control slides in the absence of primary antibodies were included for each staining. The scoring of expression of CD8+ TIL was done semi-quantitatively as described before (8). Categories for scoring were: (1) no, or sporadic cells; (2) moderate number of cells; (3) abundant occurrence of cells; (4) highly abundant occurrence of cells. Scoring was done for both, intraepithelial and stromal compartments (× 10 magnification) separately (25, 26). Scoring for p16^INK4a^ was done using a weighted score combining percentage of stained cells and the intensity of staining. Staining intensity was scored as 1+ (weak), 2+ (moderate), 3+ (intense). The fraction of stained tumor cells was scored as 1 (0–25%), 2 (26–50%), 3 (51–75%) and 4 (75–100%). The final score was generated by multiplication of both scores, leading to a score ranging from 1 to 12.

We used the median value as cut-off to dichotomize the cohort in patients either having a low (≤median) or high (>median) score. Images were acquired with the Axio Scan Z1 slide scanner using the ZEN software (Zeiss, Germany). To minimize interobserver variability, two investigators without knowledge of the clinicopathological and clinical data performed scoring. In cases of discrepancy, a final decision was made after additional examination of the specimens.

### Statistical Analysis

Differences between groups were assessed using Pearson's Chi-squared test for categorical variables, and the non-parametric Wilcoxon rank sum test for continuous variables. Correlations between continuous variables were assessed using the Spearman‘s rank correlation coefficient. Survival times were calculated from start of CRT to the date of respective events or last follow-up. Locoregional control rate (LRC) was calculated using non-complete response at first restaging or locoregional recurrence after initial complete response as event. Disease-free survival (DFS) was calculated using the date of diagnosis of locoregional failure, distant metastases, or death of any cause. Distant metastasis-free survival (DMFS) was calculated using the date of diagnosis of distant metastases or death of any cause as event. Overall survival (OS) was assessed with death of any cause as the respective event. Survival differences were visualized by Kaplan-Meier curves and the log-rank test was used for calculation. For LRC, competing risk analysis was performed using the “cmprsk” package with death as competing event.

Cut-off of CAR for DFS was calculated using the maximally selected rank statistics, which calculate the most optimal cut-off for a continuous variable regarding log-rank statistics using an established algorithm (27). The Cox proportional hazard model was used for univariate analysis to assess the influence of CAR as a continuous variable, and with the dichotomized CAR for multivariate analysis. Due to the skewness of CAR, values were transformed using the binary logarithm, leading to a broader distribution of data. Only factors found to be significant in univariate analysis were included in the multivariate one. Due to skewness of distribution, we used a binary log transformation for inclusion in cox regression models. The assumption of proportional hazard was tested by the scaled Schoenfeld residuals. Due to the low number of events, we were only able to conduct a multivariate analysis with a small set of variables and with DFS as endpoint due to the larger number of events (28). Statistical analysis was performed with R (Version 3.5) (29) using the packages maxstat, cmprsk, pROC (30) and survminer. A *p* < 0.05 was considered significant.

## Results

### Patient and Treatment Characteristics

**Figure 1:**
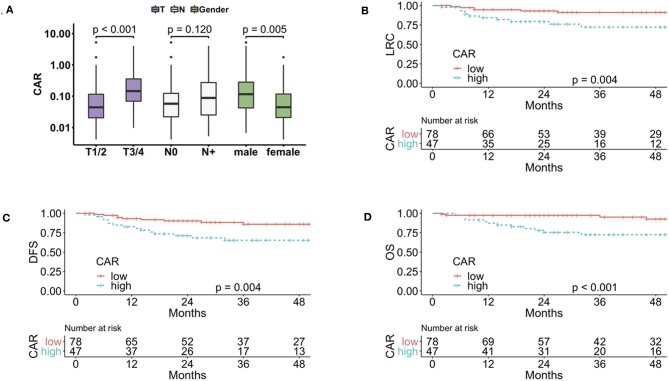
Patients with advanced T-stage and male gender had a significantly elevated CAR before initiation of treatment **(A)**. Patients with high baseline CAR had a worse LRC **(B)**, DFS **(C)**, and OS **(D)**. CAR, CRP-albumin-ratio; LRC, locoregional control; DFS, disease-free survival; OS, overall survival.

Patient and treatment characteristics are summarized in Table 1. Thirty of 125 patients (24%) were HIV-positive. Male gender (median 0.11 vs. 0.04 for females, *p* < 0.01) and advanced T-stage (0.15 for T3/4 vs. 0.04 for T1/2, *p* = 0.005) were associated with a significantly higher CAR (Figure 1A), whereas no significant association was noted for CAR with N-stage. HIV-positive patients had a significantly higher CAR (median 0.12 vs. 0.06 for HIV-negative patients, *p* < 0.001). As 28 of 30 HIV-positive patients were male, we excluded them in a subsequent analysis confirming a borderline significance for higher CAR in HIV-negative male patients compared to female patients (median 0.08 vs. 0.04, *p* = 0.053). As previous studies did not find a significant difference in the immune contexture of HIV positive and negative patients (31), and also because HIV positive patients do not have a worse prognosis under effective antiretroviral therapy (32), we did not exclude HIV positive patients in subsequent analysis. There was no correlation of CAR with age (*R* = −0.076, *p* = 0.400) or body mass index (*R* = −0.048, *p* = 0.600). Performance status was available in 100 of 126 patients (ECOG 0: 79, ECOG 1: 21) and there was no correlation with CAR in this subgroup.

**Table 1:** Patients, tumor, and blood characteristics.

		**Median (range) or *n* (%)**
Age, years		57 (34–84)
Sex	Male	58 (46)
	Female	67 (54)
HIV-Status	Positive	30 (24)
	Negative	95 (76)
T-Stage	T1	28 (22)
	T2	58 (46)
	T3	32 (26)
	T4	7 (6)
N-Stage	N0	71 (57)
	N+	54 (43)
Grading	G1	8 (6)
	G2	84 (68)
	G3	28 (22)
	Unknown	5 (4)
Pretreatment blood chemistry		
C reactive protein (mg/dl)		0.29 (0.02–21.78)
Albumin (g/dl)		4.40 (3.3–5.0)
CRP to Albumin Ratio (CAR)		0.063 (0.004–5.19)
Radiotherapy		
RT modality	3D	44 (35)
	IMRT	81 (65)
Total dose (Gy)		59.4 (50.4–64.8)

*HIV, human immunodeficiency virus; Gy, Gray; RT, radiotherapy*.

### Univariate Analysis

We used maximally-selected rank statistics to estimate the best cut-off for CAR to dichotomize the cohort of patients for the endpoint DFS. The calculated CAR cut-off was 0.117, resulting in 47 of 125 (37%) patients in the high CAR group. Using this cut-off revealed a significant association of high CAR with worse LRC (*p* = 0.005, using competing risk analysis with death as a competing risk), DMFS (*p* = 0.010), DFS (*p* = 0.004), and OS (*p* < 0.001) in univariate analysis (Figures 1B–D, Table 2). The 3 year LRC, DMFS, DFS, and OS rates were 91% vs. 72%, 93% vs. 78%, 86% vs. 65%, and 95% vs. 72% for low vs. high CAR, respectively. In a separate analysis, using the binary logarithm of CAR as a continuous variable, we found a significant association of CAR with worse DMFS (HR 1.31, *p* = 0.02), DFS (HR 1.19, *p* = 0.040), and OS (HR 1.36, *p* < 0.001) in univariate analysis (Table 2). Regarding established clinicopathologic parameters in univariate analysis, cN+ was associated with worse LRC, DMFS, and DFS, advanced T-stage was associated with worse DMFS and male gender was associated with worse DMFS and DFS (Supplementary Table S1).

**Table 2:** Results of univariate cox regression analysis with calculated cut off and binary log transformed CAR as continuous variable.

	**Dichotomized CAR**			**Binary logarithm of CAR**		
	**HR**	**95% CI**	***p*-value**	**HR**	**95% CI**	***p*-value**
LRC	3.771	1.41–10.06	**0.004**	1.181	0.97–1.44	0.1
DMFS	4.065	1.25–13.21	**0.010**	1.311	1.05–1.64	**0.02**
DFS	3.162	1.40–7.16	**0.004**	1.191	1.01–1.41	**0.04**
OS	4.99	1.80–13.92	**<0.001**	1.364	1.13–1.64	**<0.001**

*LRC, locoregional control; DMFS, distant-metastasis free survival; DFS, disease-free survival; OS, overall survival; CAR, CRP-albumin-ratio; Significant values have been marked in bold*.

**Figure 2:**
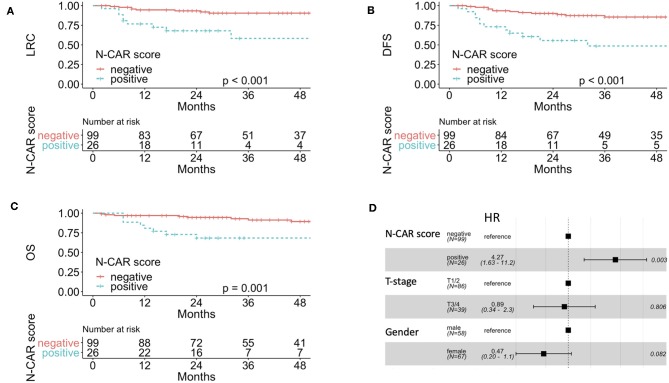
A combined score of N-stage and CAR was associated with worse LRC **(A)**, DFS **(B)**, and OS **(C)**. Forest plot showing that N-CAR remained a significant prognostic factor for DFS in a multivariate cox regression analysis with gender and T-stage **(D)**. CAR, CRP-albumin-ratio; LRC, locoregional control; DFS, disease-free survival.

In a next step, we created a combined score with CAR and N-stage as these parameters are not correlated with each other, termed N-CAR score. Only patients with both a high CAR and cN+ were considered “N-CAR positive,” whereas all other patients were scored as “N-CAR negative.” Overall, 26 of 125 patients (20.8%) were N-CAR positive. In univariate analysis the combined N-CAR score was significantly prognostic for LRC (*p* < 0.001, Figure 2A), DMFS (*p* < 0.001), DFS (*p* < 0.001, Figure 2B), and OS (*p* = 0.001, Figure 2C). The 3 year LRC, DMFS, DFS and OS rates were 91% vs. 58%, 93% vs. 66%, 86% vs. 49% and 90% vs. 68% for negative vs. positive N-CAR score, respectively. In an additional analysis, we tested our cut-off in cN0 and cN+ patients, separately. In cN+ patients high CAR remained significant for LRC (*p* = 0.04), DMFS (*p* = 0.04), DFS (*p* = 0.04), and OS (*p* = 0.01), whereas there was no significant difference in cN0 patients. Nevertheless, this has to be interpreted cautiously due to the limited number of events in cN0 patients.

### Multivariate Analysis

Due to the low number of events (LRC: 18, DMFS: 13, DFS: 25), we conducted the multivariate analysis for DFS and included CAR, gender and N-stage, as these factors were significantly associated with worse DFS in univariate analysis. In general, multivariate analysis with such a small number of event need to be interpreted cautiously. Only lymph node involvement remained a significant prognostic factor for adverse DFS (Table 3). Multivariate models for LRC, DMFS, and OS were also created but need to be interpreted cautiously due to the low number of events. Nevertheless, N-stage was the only factor that remained significant for LRC and DMFS, whereas for OS CAR remained as the only significant factor (Supplementary Table S2). As we found a correlation between T-stage and CAR, we created another model including T-Stage, CAR, and gender as variables. In this model, only CAR remained as a significant prognostic factor (HR 2.71, 95% CI 1.04–7.22, *p* = 0.042). Introduction of the N-CAR score into a multivariate cox regression model together with T-stage and gender showed that only the N-CAR score was significantly associated with DFS (Figure 2D).

**Table 3:** Results of multivariate cox regression analysis DFS using dichotomized CAR.

	**HR**	**95% CI**	***p*-value**
**DFS**			
CAR	2.13	0.91–4.99	0.082
N-stage (N+ vs. N0)	3.85	1.58–9.38	**0.003**
Gender (male vs. female)	2.18	0.91–5.21	0.079

*DFS, disease-free survival; CAR, CRP-albumin-ratio; Significant values have been marked in bold*.

### Correlation of CAR With CD8+ and p16^INK4a^

**Figure 3:**
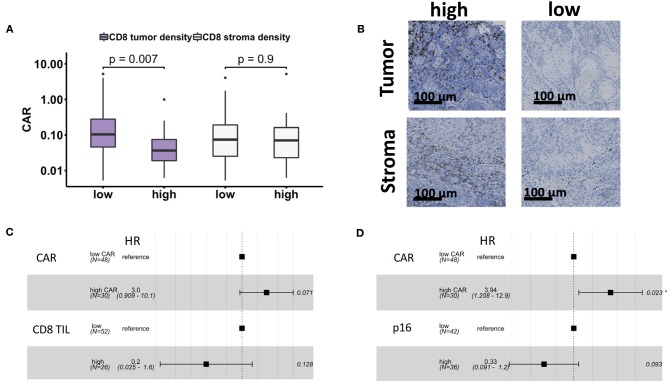
Strong intratumoral infiltration with CD8+ TIL was associated with elevated CAR at baseline, whereas there was no association between peritumoral TIL and CAR **(A)**. Exemplary stainings for high and low intratumoral and peritumoral infiltrations with CD8+ TIL **(B)**. Forest plot of a multivariate cox regression model including CAR and CD8 TIL **(C)** and CAR and p16 **(D)** for DFS. CAR, CRP-albumin-ratio; TIL, tumor-infiltrating-lymphocyte.

We measured the infiltration of tumor tissue and peritumoral stroma with CD8+ TIL in 78 patients with available tumor specimens. A high intratumoral CD8 infiltration was associated with a significantly lower CAR (*p* = 0.007), whereas there was no statistically significant association between peritumoral CD8+ TIL and CAR (*p* = 0.9, Figure 3A). No correlation between p16^INK4a^ score and CAR was observed. High intratumoral CD8+ TIL were significantly associated with a better DFS in univariate analysis (*p* = 0.03), whereas a high p16^INK4a^ score showed a trend toward better DFS (*p* = 0.06). Due to the smaller number of events in this subset of patients, we conducted two separate multivariate cox regression analysis for DFS combining one of the established molecular markers with CAR in order to avoid overfitting by including too many variables in the model (28). In combination with CD8+ TIL no variable was significant for DFS (Figure 3C), whereas CAR remained significant for DFS in the model with the p16^INK4a^ score (*p* = 0.023, Figure 3D).

## Discussion

Inflammation is one of the hallmarks of cancer (33) resulting in an exhausted state of immune cells with subsequent tumor immune escape and, thus, tumor progression (34). Chronic inflammation can, via increased production of growth factors, proangiogenic molecules and/or factors that modify the extracellular matrix, promote invasive growth and metastasis (35). CAR is a marker of inflammation and was first introduced as a prognosticator for mortality of septic patients (20). Elevated CRP-levels are associated with various inflammatory processes, whereas decreased serum albumin levels are associated with chronic diseases and malnutrition and therefore can be a result of chronic inflammatory processes that are known to play a central role in carcinogenesis (36). Thus, in the context of malignant diseases, CAR could act as a surrogate marker for prolonged chronic inflammatory processes. Recently, Kinoshita et al. (37) an association between CRP and serum albumin/malnutrition with tumor stage and prognosis has been demonstrated in various malignancies (38), but its role in ASCC remains unexplored. In the present study, we evaluated CAR as a new biomarker for patients with ASCC treated with definitive CRT. A high baseline CAR was associated with larger tumors (T3/T4), male gender, and HIV-positivity in the entire cohort. CAR was a significant prognostic marker for LRC, DMFS, DFS, and OS in univariate analysis. In multivariate analysis, only N-stage remained significant for DFS. As CAR was not correlated with N-stage in our analyses, we assessed a combined score of CAR and positive lymph nodes, termed the N-CAR score. The predictive power of this score was independent of T-stage and gender in our cohort and could be of use in future trials.

In that context, a direct functional role for CRP in tumor progression has been described in a breast cancer model, where binding of CRP to integrin alpha2 subunits on tumor cells fostered invasion (39). CRP is released by the liver upon IL-6 signaling that in turn promotes tumor progression. Interleukin 6 upregulates tumor-promoting M2 macrophages via either STAT3 phosphorylation (40) or direct inhibition of apoptosis and stimulation of proliferation, also mainly mediated by STAT3 (41). Intriguingly, an antitumor effect has been described for CRP via inhibition of epithelial-mesenchymal transition and tumor-promoting M2 macrophages in preclinical studies (42, 43). However, at present one cannot rule out that CRP has no direct effect on tumors but rather displays a down-stream effect of IL-6 mediated tumor progression.

In a subset of patients with available pretreatment tumor tissue, a strong intratumoral infiltration with CD8+ TIL was associated with lower CAR whereas no association of CAR with p16^INK4a^ immunohistochemistry as surrogate parameter for HPV infection was noted. This is in line with the notion that chronic inflammation can lead to inhibition of CD8+ TIL by various mechanisms including expansion of regulatory T-cells or upregulation of immune checkpoints like programmed death ligand 1 (PD-L1) (36). Interestingly, in renal cell carcinoma higher CRP was associated with a stronger infiltration with CD8+, FoxP3+ and CD163+ cells and subsequently with worse prognosis (44). FoxP3+ and CD163+ cells are generally regarded to be associated with immunosuppression and chronic inflammation (45, 46) and the combination with a rise in CD8+ infiltration and worse prognosis may indicate that these CD8 cells are mainly exhausted. While HPV status is an established prognostic parameter in ASCC (10) we could not find an association of CAR with p16^INK4a^.

There is no consensus regarding the optimal cut-off for CAR. Previous studies have estimated the optimal cut-off using approaches similar to ours leading to a variety of cut-offs (Table 4). The large variability in the range of cut-off values among the various studies could be explained by differences in tumor burden and degree of hypoalbuminemia. In pancreatic cancer, two studies reported different cut-offs, while the proportion of patients with metastatic disease was similar in both cohorts (24, 48). In our series in non-metastatic ASCC, only a small variation in albumin levels was observed, indicated by a small standard deviation of 0.35. Thus, the range of CAR values in our cohort were mainly a result of baseline CRP levels. Nevertheless, more variation in albumin levels may be detected in other cohorts, leading to the need for further exploration of this marker (50). Of note, we also repeated our analysis using the cut-offs in other squamous cell carcinomas (see Table 4) and found an association with worse OS using both cut-offs (Supplementary Table S3).

**Table 4:** CAR cut-offs reported in the literature.

**Author**	**Tumor entity**	**Cut-off**
Kuboki et al. (21)	HNSCC	0.32
Guo et al. (22)	Bladder	0.2
Otowa et al. (23)	Esophageal SCC	0.048
Liu et al. (24)	PDAC	0.18
Liu et al. (47)	Ovarian Cancer	0.68
Wu et al. (48)	PDAC	0.54
Zhou et al. (49)	SCLC	0.441
Kinoshita et al. (37)	HCC	0.037
Our Data	ASCC	0.117

*HNSCC, head and neck squamous cell carcinoma; SCC, squamous cell carcinoma; PDAC, pancreatic ductal.adenocarcinoma; SCLC, small cell lung cancer; HCC, hepatocellular carcinoma; ASCC, anal squamous cell carcinoma*.

The prognosis of ASCC following primary CRT varies considerably. Patients with early-stage T1-2 N0 disease, have favorable prognosis with a 3 year DFS between 80 and 90% after CRT, and such a subgroup of these patients are often overtreated with late gastrointestinal toxicity that adversely impacts quality of life (5). In contrast, the 3 year DFS is ~60% in patients with cT3-4 and /or cN+ tumors (Stage IIb-IIIC), where local and/or distant failures commonly occur (7). Accordingly, escalation strategies should be explored in these patients. In that context, CAR should be further explored as a biomarker in future trials. Such trials could involve immune modifying agents alone or in combination with CRT, with first data in the metastatic setting showing good tolerance and promising results (51, 52). Another cheap available potential predictive biomarkers are baseline hemoglobin levels (53).

There are several limitations to our study. First, the retrospective nature of our analysis could lead to selection bias and information on well-established molecular markers CD8+ TILs and p16^INK4a^ was only available for a subgroup. Second, the prognostic value of CAR as a biomarker needs to be validated in an independent cohort of patients with ASCC treated with standard CRT. Third, the optimal cut-off for CAR to stratify patients into good and poor prognostic groups needs to be established. Fourth, smoking status was not available for analysis. Fifth, performance status was only available in 100 of 126 patients and thus was not included in the analysis. Sixth, the relatively small sample size.

In conclusion, CAR is a cheap and routinely available biomarker that may be predictive of clinical outcome after CRT in ASCC. Validation in an independent cohort of patients is still needed. Further studies should aim to validate the potential of CAR as a prognostic marker in larger, preferably prospective ASCC cohorts, to help guide personalized future treatment strategies.

## Data Availability Statement

The datasets generated for this study are available on request to the corresponding author.

## Ethics Statement

The studies involving human participants were reviewed and approved by Ethics committee Goethe University Frankfurt University Hospital Frankfurt. Written informed consent for participation was not required for this study in accordance with the national legislation and the institutional requirements.

## Author Contributions

DM, FR, CR, and EF contributed to conception and design of the study and wrote sections of the manuscript. DM, FR, and PB organized the database. DM and RW performed immunohistochemical analysis. DM performed the statistical analysis and wrote the first draft of the manuscript. All authors contributed to manuscript revision, read, and approved the submitted version.

### Conflict of Interest

The authors declare that the research was conducted in the absence of any commercial or financial relationships that could be construed as a potential conflict of interest.
