# Supplementary material for: C-Reactive Protein-to-Albumin Ratio as Prognostic Marker for Anal Squamous Cell Carcinoma Treated With Chemoradiotherapy
Source: Front Oncol. 2019 Nov 8;9:1200. doi: 10.3389/fonc.2019.01200 (PMC6856140; doi:10.3389/fonc.2019.01200)
Supplement: Supplementary file 1 [file Table_1.DOCX]

Supplementary Material

# Supplementary Tables

**Supplementary Table S1**. Results of univiariate analysis

|  | LRC  p value | DMFS  p value | DFS  p value | OS  p value |
| --- | --- | --- | --- | --- |
|  |  |  |  |  |
| Male Gender | 0.070 | **0.020** | **0.020** | 0.2 |
| T3/4 | 0.400 | **0.002** | 0.100 | 0.06 |
| N+ | **0.004** | **0.005** | **< 0.001** | 0.2 |
| High CAR | **0.004** | **0.010** | **0.004** | **< 0.001** |

Abbreviations: LRC, locoregional control; DMFS, distant-metastasis-free survival; DFS, disease-free survival; OS, overall survival; Significant p values marked bold.

**Supplementary Table S2** Results of multivariate analysis including dichotomized CAR, N-stage and Gender for LRC, DMFS and OS.

|  | HR | 95% CI | p-value |
| --- | --- | --- | --- |
| **LRC** |  |  |  |
| CAR | 2.70 | 0.98 – 7.45 | 0.054 |
| N-stage (N+ vs. N0) | 3.58 | 1.25 – 10.26 | **0.018** |
| Gender (male vs. female) | 2.04 | 0.74 – 5.62 | 0.166 |
| **DMFS** |  |  |  |
| CAR | 2.28 | 0.66 – 7.81 | 0.189 |
| N-stage (N+ vs. N0) | 4.49 | 1.20 – 16.80 | **0.026** |
| Gender (male vs. female) | 3.42 | 0.91 – 12.88 | 0.069 |
| **OS** |  |  |  |
| CAR | 4.47 | 1.53 – 13.03 | **0.006** |
| N-stage (N+ vs. N0) | 1.29 | 0.51 – 3.25 | 0.584 |
| Gender (male vs. female) | 1.26 | 0.48 – 3.33 | 0.638 |

Abbreviations: LRC, locoregional control; CAR,CRP – albumin ratio; DMFS, distant-metastasis-free survival; OS, overall survival; Significant p values marked bold.

**Supplementary Table S3**. Results of univariate analysis according to already published cut-offs for CAR in esophageal squamous cell carcinoma and head and neck squamous cell carcinoma. LRC, DMFS and DFS were calculated using competing risk analysis.

|  | LRC  p value | DMFS  p value | DFS  p value | OS  p value |
| --- | --- | --- | --- | --- |
| Kuboki et al. (21) | 0.7 | 0.06 | 0.2 | **< 0.001** |
| Otowa et al.(23) | 0.3 | **0.04** | 0.09 | **0.02** |

Abbreviations: LRC, locoregional control; DMFS, distant-metastasis-free survival; DFS, disease-free survival; OS, overall survival; Significant p values marked bold.
